# Reversing Hemianopia by Multisensory Training Under Anesthesia

**DOI:** 10.3389/fnsys.2020.00004

**Published:** 2020-01-31

**Authors:** Huai Jiang, Benjamin A. Rowland, Barry E. Stein

**Affiliations:** Department of Neurobiology and Anatomy, Wake Forest School of Medicine, Medical Center Boulevard, Winston-Salem, NC, United States

**Keywords:** multisensory, rehabilitation, vision, cross-modal, hemianopia

## Abstract

Hemianopia is characterized by blindness in one half of the visual field and is a common consequence of stroke and unilateral injury to the visual cortex. There are few effective rehabilitative strategies that can relieve it. Using the cat as an animal model of hemianopia, we found that blindness induced by lesions targeting all contiguous areas of the visual cortex could be rapidly reversed by a non-invasive, multisensory (auditory-visual) exposure procedure even while animals were anesthetized. Surprisingly few trials were required to reinstate vision in the previously blind hemisphere. That rehabilitation was possible under anesthesia indicates that the visuomotor behaviors commonly believed to be essential are not required for this recovery, nor are factors such as attention, motivation, reward, or the various other cognitive features that are generally thought to facilitate neuro-rehabilitative therapies.

## Introduction

Extensive damage to the visual cortex on one side of the brain produces blindness in the opposite hemifield (hemianopia) despite the sparing of other visual centers far from the site of the physical insult (Sand et al., 2013; Goodwin, 2014). Of special note is the superior colliculus (SC), a midbrain structure that plays a major role in detecting, localizing, and orienting to visual targets. Its multisensory neurons allow it to use non-visual cues to facilitate this process (Stein and Meredith, 1993), and its location in the midbrain ensures that it is not directly damaged by a hemianopia-inducing cortical insult. Yet, as shown in the cat model of hemianopia, the loss of visual responses in the multisensory layers of the SC and the total absence of visual detection and orientation responses to contralateral visual stimuli following lesions of visual cortex reveal that it too is compromised, presumably *via* secondary excitotoxic injuries that may alter other input structures such as the basal ganglia (Jiang et al., 2009, 2015). Interestingly, the dysfunction of SC appeared to be limited to its visual role. Its other sensory representations and sensorimotor roles remained intact: SC-mediated auditory and tactile detection and orientation responses were readily elicited (see also Sprague and Meikle, 1965).

Previously it was shown that hemianopia could be reversed using a non-invasive multisensory training paradigm (Jiang et al., 2015). The procedure consisted of presenting cross-modal combinations of spatiotemporally congruent auditory-visual cues in the blind hemifield of alert animals engaged in a sensory localization task. Because the animals were not deafened by the cortical lesion, they readily responded to the auditory-visual stimulus complex. After only a few weeks of daily multisensory training sessions, a striking change occurred: not only could the animals now detect and localize a visual stimulus throughout the previously blind hemifield, but they could also discriminate elementary visual patterns there. Visual responses that had been lost in the multisensory layers of the ipsilesional SC also returned, and cortico-SC circuits normally engaged in multisensory integration (i.e., projections from the anterior ectosylvian sulcus, AES) were found to be crucial for the recovery. The recovery could not be induced by training with visual or auditory cues alone. In an important series of studies in human patients, Làdavas and colleagues (Bolognini et al., 2005; Leo et al., 2008; Passamonti et al., 2009; Dundon et al., 2015a,b) used a similar training paradigm and also met with success in evoking contralesional visual responses.

It is commonly believed that the success of this rehabilitative paradigm is a retraining of the visuomotor targeting behavior itself (see, review in Dundon et al., 2015a). In this case, the key factor would be the orienting action (initially elicited by the auditory stimulus) in the presence of the visual stimulus. Also, if true, it is reasonable to hypothesize that the effectiveness of this paradigm would be facilitated by other factors such as motivation, attention, arousal, and reinforcement, as these are commonly believed to enhance most neuro-rehabilitative therapies. An alternative explanation, however, is that the paradigm operates *via* the brain’s inherent mechanisms for multisensory plasticity, which operate independent of these factors and can be engaged under anesthesia (Yu et al., 2013). In this case, the requirement would only be repeated, reliable exposure to the visual-auditory stimulus complex in the blinded hemifield. The present study examined this possibility directly.

## Materials and Methods

Adult mongrel cats (four male, three female) were obtained from a USDA-licensed commercial animal breeding facility (Liberty Labs, Waverly, NY, USA). The experimental procedures used were in compliance with the National Institutes of Health “Guide for the Care and Use of Laboratory Animals” (8th edition, NRC 2011) and approved by the Institutional Animal Care and Use Committee at Wake Forest School of Medicine. Each animal was first screened to ensure that it was tractable and responded to visual and auditory stimuli in both hemifields. All efforts were made to minimize the number of animals used.

### Visual Detection and Orientation Testing

**Figure 1:**
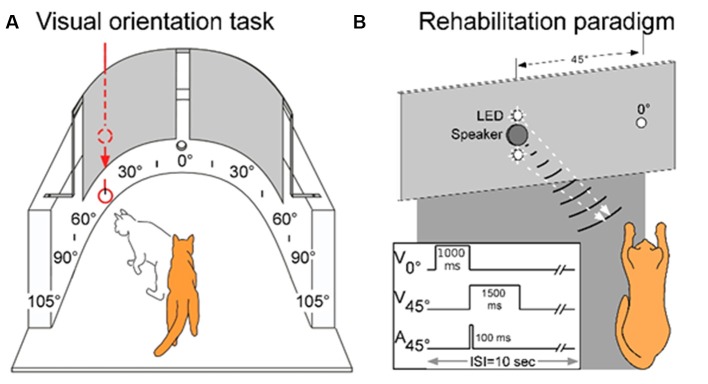
The testing, training, and multisensory exposure paradigms. **(A)** Visual and auditory detection/localization capabilities were first assessed on both sides of space using a simple behavioral task. Cats were trained to fixate forward at 0° then orient to, and directly approach, a visual or auditory stimulus at any location in space. Visual stimuli were produced by lowering a ping pong ball below an obscuring curtain, and auditory stimuli were produced by tapping the ball against the apparatus wall while still obscured by the curtain. **(B)** Following surgery, a rehabilitation paradigm consisted of weekly sessions in which animals were exposed to cross-modal cues while anesthetized. As shown by the schematic at the lower left, the central LED (at 0°) of the display was briefly illuminated to signal the onset of the trial. It was followed by the combined LED-broadband noise burst at 45° in the contralesional hemifield. Traces illustrate the onset and duration of the stimuli. Panel **(A)** adapted from Jiang et al. (2015).

Visual orientation capabilities were quantitatively evaluated in a semicircular perimetry arena using previously described methods (see Jiang et al., 2015, see also Figure 1A). Animals were maintained at 80%–85% of body weight and obtained most of their daily food intake during, or immediately after, each behavioral session. Each animal was first trained to fixate directly ahead at a food reward held in forceps by one experimenter and protruded through a hole in the front wall of the apparatus 58 cm ahead at the 0° fixation point. Trial initiation was always contingent upon the animal establishing fixation. Once released by the animal handler (a second experimenter), the animal was required to move directly ahead to obtain the food reward. It was then trained to respond to the test stimulus (a white ping-pong ball at the end of a stick) presented at any 15° interval from 105° left to 105° right. This required little training as animals responded to the stimulus almost reflexively. Stimuli were presented manually and introduced suddenly from behind a black curtain while the animal was fixating. Additionally, on some trials, the ball remained hidden behind the opaque curtain and was tapped on the side of the apparatus to produce an auditory stimulus. If the animal oriented to and approached any test stimulus it was rewarded there, but could also move directly ahead to obtain a similar reward at the fixation point. The animal handler did not know the location of the upcoming test stimulus. This was determined by the experimenter holding the food reward, who also ensured that the trial did not begin if the animal had broken fixation. The verbal command “Go” triggered the release of the animal. “Catch trials” in which no stimulus was presented were interleaved with test trials at different locations to encourage the animal to minimize breaks in fixation, scanning movements, and “false” responses. Generally, in a given session, each of the 15° locations was tested at least 4–5 times. With few exceptions, the total number of trials/location was at least 100. The training criterion was an average of 95% correct responses. All animals reached criterion readily, had normal visual fields, and their weekly weight records revealed stable weight profiles.

### Visual Cortex Ablation

**Figure 2:**
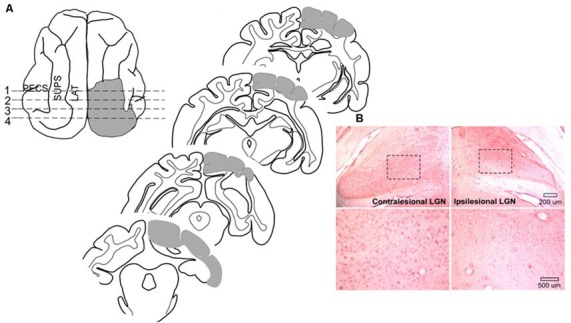
Cortical areas ablated and degeneration of the Lateral Geniculate Nucleus (LGN). **(A)** Tracings of the brain regions ablated in one animal showing both the dorsal view and coronal sections. Ablated areas are indicated in gray. All animals received similar lesions. All lesions induced a profound contralesional hemianopia. **(B)** Microscopic images of the LGN in this animal. Zoomed images of the outlined area below reveal the near complete absence of large neurons in the LGN on the same side of the brain (ipsilesional).

Surgical procedures were conducted using sterile techniques. Animals were sedated with an initial injection of buprenorphine (0.005–0.01 mg/kg, i.m.) /acepromazine (0.05–0.1 mg/kg, i.m.) to render them tractable. Then, each animal was anesthetized with sodium pentobarbital (22–30 mg/kg, i.v; Jiang et al., 2009, 2015). Antibiotics (cefazolin, 20–30 mg/kg, i.m.) were provided preoperatively and, after the loss of reflexes to external stimuli, the animal was intubated through the mouth for later ventilation and placed in a stereotaxic head-holder and on a heating pad. A cannula was placed in the saphenous vein, and body temperature, expiratory CO_2_, blood pressure, and heart rate were monitored *via* a SurgiVet Advisor (Smith Medical, Dublin, OH, USA) and maintained within normal physiological limits. The hair over the surgical site was removed and the area was coated with betadine. The scalp was opened, a craniotomy was made, the dura was reflected and the gray matter was aspirated. The lesion (see Figure 2) was made in the left hemisphere of one animal and the right hemisphere of six animals (prior work by Jiang et al., 2015 shows no hemispheric differences) to include the posterior three-fourths of the lateral and suprasylvian gyri, a portion of the posterior ectosylvian gyrus, the medial aspect of the cortex posterior to the cruciate gyrus above the splenial sulcus so that the lesion targeted Brodmann areas: 17, 18, 19, 20a, 20b, 21a, 21b, 5, 7, and the DLS, VLS, PS, PMLS, PLLS, AMLS, ALLS, and SVA, but always spared the AES. This large lesion causes degeneration of the ipsilesional lateral geniculate nucleus (LGN; Figure 2).

The lesion cavity was filled with moist gel foam, the cranial bone was replaced, and the incision closed with sutures. A corticosteroid anti-inflammatory (dexamethasone; 1 mg/kg, i.m.) was given immediately after surgery to control edema, and analgesics (buprenorphine 0.005–0.01 mg/kg, i.m.) were routinely administered for a minimum of 24 h after surgery, and then provided daily as needed. The antibiotic cefazolin (20–30 mg/kg, i.m.) was given after surgery and continued as needed. Saline (50–200 ml, s.q. or i.v.) was provided to compensate for fluid loss. After the return of sternal recumbence and active locomotion, the animal was returned to its home cage.

Following surgery, each animal exhibited the characteristic tonic head deviation toward the lesion side and ipsiversive circling behavior that is associated with these lesions (Sprague, 1966; Sherman, 1977; Jiang et al., 2015). These symptoms resolved within 1 or 2 days. However, the absence of orientation to all manually presented visual stimuli and the total absence of blink-to-threat reflexes to contralesional visual stimuli persisted for the 3 month period used as the criterion for a “permanent” visual defect. The animal was then implanted with a head holding device to be used during rehabilitative training.

### Animal Preparation for Multisensory Exposure and Electrophysiological Recording

Surgical procedures similar to those described above were used here. However, in this case, anesthesia was induced with ketamine hydrochloride (20–30 mg/kg, i.m.) and acepromazine maleate (0.05–0.1 mg/kg, i.m.), and maintained by artificial ventilation with isoflurane (0.5–4.0%). Expiratory CO_2_ was maintained at 3.5%–4.5%, eyes were covered with a topical ophthalmic ointment and the craniotomy was made to provide access to the SC on both sides of the brain. A stainless-steel recording well/head-holder was fitted over the craniotomy and anchored to the skull with stainless-steel screws and dental acrylic (McHaffie and Stein, 1983). After a 10–14 day recovery period, multisensory rehabilitative exposures began.

During each of the exposure or recording sessions the animal was anesthetized in its home cage with ketamine hydrochloride (20–30 mg/kg, i.m.) and acepromazine maleate (0.05–0.1 mg/kg, i.m.). It was then transported to the experimental room. An endotracheal tube was inserted, and the animal was artificially respired. Its head was secured by attaching the head-holder to the stereotaxic frame without wounds or pressure points, and paralysis was induced with pancuronium bromide; (0.1 mg/kg, i.v.) to fix the eyes and pinnae. During the multisensory exposure period anesthesia, paralysis, and hydration were maintained *via* continuous intravenous infusion of ketamine hydrochloride (5–10 mg/kg/h) and pancuronium bromide (0.04–0.1 mg/kg/h) in 5% dextrose Ringer’s solution (3–6 ml/h) through the saphenous vein. Blood pressure, heart rate, SpO_2_ and respiratory CO_2_ level were monitored continuously (Digital Vital Signs Monitor, SurgiVet V9200). End-tidal CO_2_ was maintained at 3.5–4.5%. SpO_2_ was maintained at >90%. Body temperature was kept at 37–38°C using a heating pad. The pupils were dilated with ophthalmic atropine sulfate (1%), and the eyes were fitted with contact lenses to focus them and prevent corneal drying.

### The Multisensory Exposure Paradigm

Previous results have shown that extensive, repeated exposure to modality-specific visual or auditory stimuli in the blinded hemifield did not rehabilitate hemianopia, nor did exposure to visual-auditory pairs that were spatially or temporally incongruent (Jiang et al., 2015; Dakos et al., 2019a,b). Thus, all training trials contained spatiotemporally congruent visual-auditory stimulus pairs. The exposure sessions were conducted once/week and were preceded by a 2 h period of adaptation in a darkened room. In each exposure session, a pair of spatiotemporally congruent auditory-visual stimuli was repeatedly presented at 6-s intervals at 45° in the contralesional hemifield (Figure 1B). One animal was also tested with a series of auditory-visual exposures in the ipsilesional hemifield. The visual stimulus was presented for 1,500 ms against a dark background (~0.75 cd/m^2^). It was composed of a vertically-displaced pair of 10 mm white LEDs (the speaker for auditory stimulus delivery was located between the two LEDs), each covered by a diffusing filter made from a section of a white ping-pong ball (~13.8 cd/m^2^). The diameter of each light circle produced was approximately 5°. The center of the top circle was elevated approximately 2° above the animal’s eyes and the bottom circle was approximately 7° below its eyes. The auditory stimulus was a broadband noise burst, 75 dB SPL against an ambient background of 48.4–52.7 dB SPL. It was presented for 100 ms. Animals received between 100 and 2,400 auditory-visual exposures per session. In one animal the number of stimulus presentations was varied in each session, in all others it was fixed at either: 100, 600, or 2,400 exposures/session.

### Probing for Visual Recovery During the Multisensory Exposure Period

Rehabilitative success was assessed with “probe” trials in the perimetry arena (Figure 1A) on days in which there was no exposure session. These involved detecting the visual stimulus (the white ping-pong ball) at each of the target locations (see “Visual Detection and Orientation Testing” section above). Anesthetized exposure sessions were stopped at the first sign that contralesional visual orientation capabilities were restored. At that point, only behavioral assays were continued for a minimum of 2 months to verify the extent and persistence of the recovered function.

### Electrophysiology Procedure

Animals were prepared for recording sessions (see above) after behavioral tests were completed. The purpose of these recordings was to determine whether visually-responsive neurons could be identified in the intermediate and deep layers of the SC of rehabilitated animals. Previous studies determined that this visual responsiveness is lost consequent to the lesions performed here and that it is restored concomitant with the return of the visually-guided behaviors tested here. Neuronal activity was recorded extracellularly with epoxylite-insulated tungsten microelectrodes (2–4 MΩ), then were bandpass-filtered, amplified, displayed on an oscilloscope, and subsequently processed to computer disc using a 1401+ hardware acquisition system (CED Systems, Cambridge, England). The single neuronal activity was sorted by running with CED Spike2 software. The visual receptive fields of isolated SC neurons were mapped on a Plexiglas hemisphere using a moving or stationary spot or bar of light from a hand-held ophthalmoscope. The presence of auditory responsiveness overlapping visual receptive fields was determined using broadband noise bursts (100 ms duration, 70 dB SPL) delivered from a movable speaker. For all quantitative tests, visual and auditory stimuli were presented repeatedly (*n* = 10–20 times/test, at 6–10 s intervals). Visual stimuli of various shapes and sizes were presented through an electronically-controlled, galvanometer-driven mirror system. Auditory stimuli were controlled by a custom-built audio generator. The magnitudes of responses to visual and auditory stimuli presented alone and together in spatiotemporal concordance (i.e., simultaneously) were quantified as the mean number of stimulus-elicited impulses. Multisensory enhancement (ME) was quantified as the percent difference between the response elicited by a visual-auditory pair and the most robust response elicited by one of the component stimuli (Meredith and Stein, 1983).

### Statistical Methods

All data are illustrated with the lesion depicted on the right side and the left side of space as contralesional. Data were analyzed for the central 180° of visual space in which pre-lesion visual performance was most reliable in all animals. Final stimulus detection and orientation accuracy were assessed with *X*^2^ tests. Logistic regression was used to determine whether any significant differences in visual recovery were related to the different multisensory training conditions. This was accomplished by comparing regression models fit to data pooled across all animals (using nearest-neighbor interpolation to fill gaps) to models in which a single animal was extracted and fit separately from the pool. This allowed each animal’s performance to be compared to the pooled data. The difference in deviances between the two models was evaluated against an *X*^2^ distribution with 1 degree of freedom. The significance of physiological responses was evaluated with two-tailed *t*-tests.

### Histological Evaluation of Cortical Lesions

At the termination of experimentation each animal was sedated with ketamine hydrochloride (20–30 mg/kg, i.m.) and acepromazine maleate (0.05–0.1 mg/kg i.m.) and, following the loss of reflexes, given a lethal dose of pentobarbital (100 mg/kg; i.p.). It was then perfused transcardially with 0.9% saline followed by 4% paraformaldehyde. The brain was removed, cut on a cryostat, and processed using routine histological procedures (neutral red staining; see Figure 2). The cortical lesion was reconstructed from photographs of the tissue block on the cryostat while serial coronal sections were taken. These were then referenced to standard anatomical maps (Scannell et al., 1996). Retrograde degeneration of the dorsal LGN was visualized microscopically.

## Results

### Behavioral Results

**Figure 3:**
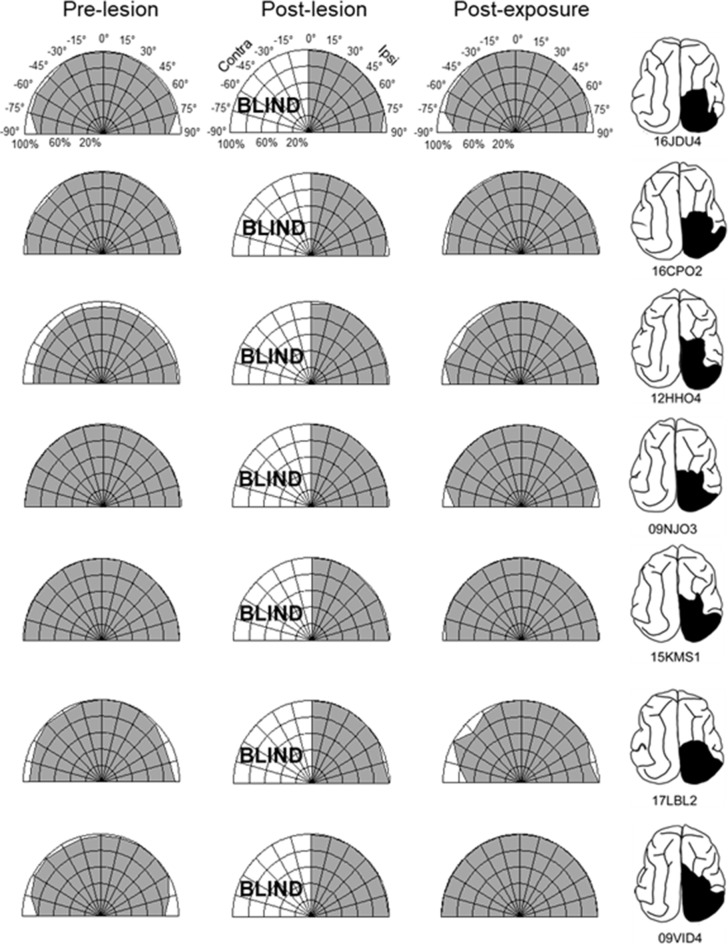
Visual detection and orientation capabilities for each animal before the lesion (pre-lesion column), >3 months after the lesion (post-lesion), and after multisensory exposure (post-exposure). Polar charts depict the accuracy of responses to visual stimuli presented at eccentricities between 90° to the left and right of fixation (15° intervals). Concentric circles in the plot indicate accuracy increments of 20%. Performance of all animals at all locations was near-perfect prior to the lesion. After the lesion, responses to contralesional visual stimuli disappeared (“BLIND”), but returned to near-perfect following multisensory exposure. For illustration the contralesional hemifield is always drawn on the left.

**Figure 4:**
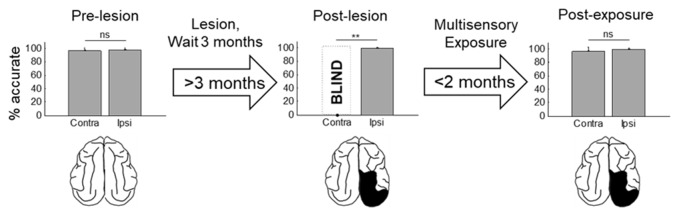
Summary of the population results. Plotted is the percentage of accurate responses to visual stimuli averaged across animals and locations on the side of space ipsilateral (ipsi) and contralateral (contra) to the visual cortex lesion at three different time points: before the lesion (pre-lesion), >3 months after the lesion but before beginning the multisensory exposure series (post-lesion), and after the series was complete and behavior had stabilized (post-exposure, this required exposure sessions over several weeks). Error bars indicate the standard deviation across animals. Note that the results are highly consistent across animals. Dashed bar labeled “BLIND” indicates that, after the lesion and prior to rehabilitation, none of the animals detected visual stimuli at any tested location in contralesional space. Exemplar schematics of the lesion accompany each plot for illustration. ns, not significant, **p < 0.001.

The repeated presentation, to anesthetized animals, of congruent auditory-visual stimuli in the hemianopic field proved to be effective in rehabilitating hemianopia. Prior to the lesion, every animal achieved near-perfect performance in detecting visual stimuli at each tested location in the central 180° (Figures 3, 4, “pre-lesion”). However, testing after a week of post-surgical recovery revealed that responses in contralesional space were entirely eliminated. Responses to visual stimuli in the ipsilesional (i.e., normal) hemifield remained intact, as did responses to auditory stimuli in both hemifields.

The hemianopia in each of these animals persisted throughout the post-surgical, pre-intervention period, which was a minimum of 3 months and in one case was extended to 15 months. After establishing that the defect was stable (Figures 3, 4, “post-lesion”), multisensory exposure sessions began. In each case, the animal was anesthetized and paralyzed. After 3–7 weeks of these sessions, all animals began responding to visual stimuli in contralesional space. The delay between the start of the exposure sessions and the first signs of recovery was related to the density of exposures per session, but not systematically. There were no significant differences in the timing of recovery onset for animals given 600 exposures/session (range: 3–4 weeks) vs. 2,400 exposures/session (4 weeks; Wilcoxon test on days-until-recovery: *p* = 0.63), despite the four-fold increase in exposure density. However, reducing the density of exposures/session to 100 doubled the recovery period to 7 weeks. The timeline of six animals’ exposure and recovery is described in Table 1. The table shows the total number of auditory-visual exposure trials, the number of exposure sessions, weeks of exposure, and the number of days between the start of the recovery, as well as when visual detection/localization performance had achieved pre-lesion levels. Data from the pilot animal (09NJO3) are not included despite its recovery, because cross-modal stimulus exposure was not systematic during this initial exploratory study (varying in frequency, timing, location, and identity).

**Table 1:** Exposure and recovery timelines for six animals (pilot animal excluded, see text).

Animal name	Exposures/Week	Exposures until the first contralesional response	Days from first contralesional response to full recovery
09VID4	600	2,400 (4 weeks)	6
12HHO4	600	3,000 (5 weeks)	7
15KMS1	2,400	12,000 (5 weeks)	10
16JDU4	100	800 (8 weeks)	5
16CPO2	600	2,400 (4 weeks)	7
17LBL2	600	3,000 (5 weeks)	8

*The leftmost column indicates animal identity. The second column reports the number of weekly exposures. The third column shows the total number of auditory-visual exposures (and a number of weekly sessions), prior to the first response to a contralesional visual stimulus. The last column indicates the number of days between that first contralesional response and visual performance reaching pre-lesion levels. Each animal was tested with a 2–5 visual test/trials/location/day*.

**Figure 5:**
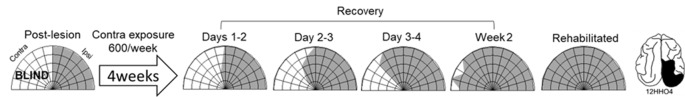
Visual responsiveness recovered in a central-to-peripheral pattern. A hemianopic animal remained blind in contralesional space after 4 weeks of exposure sessions (600 auditory-visual exposures/session). Contralesional visual responses were first observed in the 5th week at 15°. The effective region expanded to 45° within several days (Day 0 was a day of exposure, and the results depicted were averaged over 2 days), and to at all contralesional locations tested within 2 weeks.

Exposure sessions for each animal were terminated at the first appearance of contralesional visual responses. These initial responses always appeared at a location in central visual space (i.e., 15° or 30°, see Figure 5). The effective region expanded thereafter to more peripheral locations. However, within 1–2 weeks every animal reached ceiling performance at every contralesional location tested (Figures 3, 4, “post-exposure”). This central-to-peripheral pattern was previously observed in animals rehabilitated while awake (Jiang et al., 2015), and occurred despite the fact that auditory-visual exposure stimuli were presented only at 45° in contralesional space. Apparently, the exposure location neither specifies the location at which responses will first be observed nor the extent of the restored visual field. The paradigm does not require exposure at every location to be successful. Furthermore, visual restoration did not vary systematically by exposure density and, except for the animal with 100 exposures/sessions, all animals recovered within approximately the same time period.

**Figure 6:**
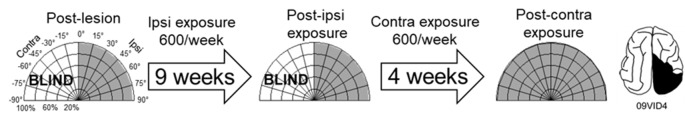
Multisensory training in the unaffected (ipsilesional) hemifield failed to induce recovery from hemianopia. Nine weeks of ipsilesional multisensory exposure at 45° (600 exposures/session) failed to ameliorate the animal’s hemianopia (conventions are the same as in Figure 4). At week 10 the cross-modal stimulus was moved to 45° in the blind (contralesional) hemifield. A series of exposures (600/session) at this location led to visual recovery within the same time frame as in animals that only had multisensory exposure in the blind hemifield.

As a control, one animal was exposed to cross-modal stimuli (600 exposures/session for 9 weeks) at the homotopic location in the unaffected (ipsilesional) hemifield. These exposures failed to induce recovery. The animal was then switched to 600 exposures/session at 45° in the blind (contralesional) hemifield. It recovered at almost the same rate, and with the same pattern, as did its counterparts whose training began in the blind hemifield (Figure 6).

*X*^2^ tests showed that each animal’s visual detection and localization performance showed no significant deficit in the rehabilitated hemifield (from 15° to 90° of eccentricity, *p*-value range: 0.998–1, DF = 6). There was also no performance difference from that in ipsilesional space (*p*-value range: 0.993–1, DF = 6), or from pre-lesion performance (*p*-value range: 0.990–1, DF = 6).

### Electrophysiological Results

In the previous Jiang et al. (2015) study, it was noted that the superficial SC layers of hemianopic animals remained rich in visually-responsive neurons and that some visually-responsive neurons were also spared in the deeper, multisensory, layers of the SC. However, the spared deeper layer visual neurons had receptive fields that were restricted to central space (<15° from the midline). These neurons, which receive visual inputs directly from the retina and indirectly from extrastriate cortex in both hemispheres, play an important role in fixation (Baleydier, 1977; Baleydier et al., 1983; Ogasawara et al., 1984; Guitton and Munoz, 1991; Meredith and Ramoa, 1998) which was maintained in these animals. In contrast, the deep layer neurons that lost their visual responsiveness were those with more peripheral (e.g., >15° or more) receptive field centers, and which play a role in contralateral visuomotor responses (Stein and Clamann, 1981; Sparks, 1986; Jay and Sparks, 1987; Sparks and Hartwich-Young, 1989; Guitton and Munoz, 1991; Paré et al., 1994). This is consistent with the lost visual function observed here.

To determine whether visual responsiveness was also present in these deep layer neurons following rehabilitation under anesthesia, the same electrophysiological recording procedures used by Jiang et al. (2015) were conducted here in three animals.

**Figure 7:**
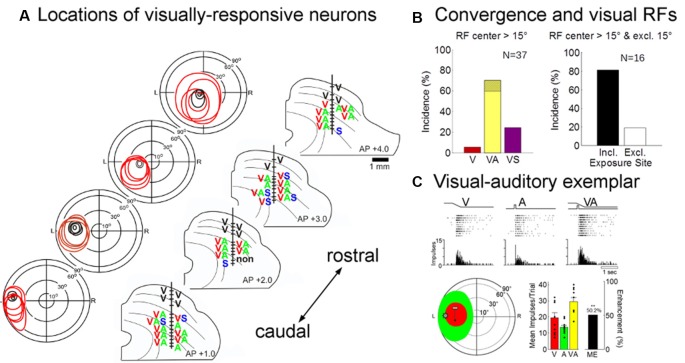
Physiological recordings from the ipsilesional superior colliculus (SC) in an exemplar animal after rehabilitation. **(A)** Electrode penetrations were made in this animal at several different anterior-posterior positions in order to span the region in which visual responsiveness was lost (i.e., beyond the central 15°). The visual receptive fields, location, and modality convergence pattern for isolated neurons in these penetrations are indicated by letters (V, visual; A, auditory; S, somatosensory). Unisensory visual neurons are shown in black (note that all superficial layer visual neurons are unisensory). As in the normal SC, deeper layer visual receptive fields are far larger than their superficial counterparts, but maintain general spatial alignment with them. Note that almost all visually-responsive neurons in the region of interest were overtly multisensory. (**B**; Left) The population data show that a large proportion of the visually responsive neurons in the reactivated region were also overtly responsive to auditory stimuli (note that the hatched region on yellow bar shows that was also responsive to somatosensory stimuli, VAS). (Right) The receptive fields of these “recovered” neurons typically encompassed the 45° exposure site (incl) without encroaching on central space (15°). **(C)** The multisensory responses of one of these visual neurons are illustrated here in rasters and peristimulus time histograms at the top. The neuron’s multisensory receptive fields are shown just below (red = V, green = A; icons show stimulus positions). Summary histograms (lower right) show the neuron’s responses to the V and A stimuli individually and the multisensory enhancement (ME = 50.2%) evoked by their combination (**p < 0.01, 2-tailed *t*-test). Overlaid dot plots show response magnitude on each trial. Error bars indicate standard error of the mean.

Eighty-five neurons were recorded in the multisensory layers of the ipsilesional SC (*n* = 46 from an animal given 600 exposures/session, *n* = 29 from the animal given 100 exposures/session, and *n* = 10 from the animal given 2,400 exposures/session). Visually-responsive neurons were readily found in each of these animals (no inter-animal differences were observed, see exemplars in Figure 7A), and their pooled modality convergence patterns are shown in the left plot of Figure 7B. Many (74%, 58/78) of the visual receptive fields recorded in rehabilitated animals were very large and extended into central visual space. An overwhelming majority of these neurons were also overtly responsive to auditory inputs. The incidence of visually-responsive neurons also sensitive to the auditory modality (70%, 26/37) was roughly twice that expected given their incidence in the normal SC and in recordings from the contralesional SC of hemianopic animals (Meredith and Stein, 1986; Jiang et al., 2015). Given the high incidence (>30%) of “covert” multisensory neurons (one of the inputs is subthreshold, see Yu et al., 2013), this is likely to be an underestimate of neurons receiving an auditory input.

These data are consistent with the suggestion that the restoration of visual responsiveness depends on the cooperative interactions of convergent visual and auditory inputs onto the same neurons. Indeed, all but three (13/16) of the visually-responsive neurons in the recovered visual hemifield (receptive fields >15° of eccentricity) had their visual (and auditory) receptive fields encompassing the training site (45°; Figure 7B, right). The visual responses of these neurons were often modulated by auditory stimuli. In only a few cases was their ability to integrate cross-modal cues tested systematically, but this ability is a characteristic feature of multisensory neurons in the normal SC and was clearly evident in the exemplar presented in Figure 7C. Its visual-auditory response was significantly elevated above that elicited by either modality-specific component stimulus.

## Discussion

That visual responsiveness was restored in hemianopic animals by exposure to auditory-visual stimuli while they were anesthetized and paralyzed reveals that overt visuomotor behavior is not a requirement in this context, nor are any of the organismic variables that typically play important roles in learning and in rehabilitative therapies: e.g., alertness, explicit reward, engagement in the task, and many cognitive and motivational factors. Although this may seem surprising, it is consistent with work demonstrating similar visuomotor “recovery” after surgical intervention to remove sources of inhibitory influence from the intact hemisphere (Sprague and Meikle, 1965; Sprague, 1966; Sherman, 1977; Wallace et al., 1989, 1990; Lomber and Payne, 1996; Lomber et al., 2002).

The multisensory rehabilitation paradigm has also been shown to restore some visual responsiveness in human hemianopic populations (Bolognini et al., 2005; Dundon et al., 2015a; see also Purpura et al., 2017). However, some differences in the results have also been noted. Rehabilitated cats appear capable of extensive visual processing, including rudimentary pattern discrimination in the previously blind hemifield (Jiang et al., 2015). This strongly suggests that they are aware of those visual events. But rehabilitated human patients, despite being able to respond to visual stimuli in the previously blind hemifield, report a lack of awareness of those visual events. This may reflect a species difference, but may also reflect significant procedural differences. The absence of visual awareness in rehabilitated patients was concluded based on their reports when required to maintain fixation during visual stimulus presentation (i.e., they suppressed orientation responses). Given that visual responses to peripheral stimuli can also be suppressed in such a paradigm (including in the SC, e.g., see Rensink et al., 1997; Simons and Levin, 1997; Meredith and Ramoa, 1998), probably, the lack of visual awareness as a consequence of how the task constraints impacted the circuit. This possibility remains to be examined experimentally.

The anesthetized cross-modal exposure paradigm utilized here may not be a viable option for human patients; however, the current observations provide essential insights into the underlying process of recovery. Of particular interest is that the present findings, combined with the fact that exposure to auditory-alone or visual-alone stimuli are both ineffective in this rehabilitation (Jiang et al., 2015), strongly support the conclusion that recovery does not rely on merely drawing attention to the compromised hemifield. Rather, it appears to depend on mechanisms of multisensory plasticity that are engaged by cross-modal stimuli in the compromised hemifield. Violating the spatial or temporal requirements for SC multisensory integration in this paradigm also renders it ineffective in rehabilitation (see Dakos et al., 2019a,b). Repeated auditory-visual stimulation in the intact hemifield is ineffective in restoring vision, and had no salutary effect on subsequent multisensory exposure.

It is interesting to note how little experience with cross-modal stimuli was needed to induce recovery. In the paradigm, stimulus exposures were provided every 6 s. Thus, animals rehabilitated by 600 exposures/session were given only 1 h of exposure per week, yet were recovered in approximately 4 weeks (4 h of exposure in total). Neither the speed of recovery nor its extent was significantly facilitated when the number of exposures was quadrupled from 600 to 2,400. When the number of exposures/session was reduced to 100, and exposure sessions only lasted for 10 min per week, recovery was initiated after 8 weeks (80 min of exposure in total). In both cases, the exposures represented a very small amount of the animals’ total sensory experience during the rehabilitation period. These observations underscore the power that statistically regular sensory exposure has to reshape neural processing dynamics (see also Xu et al., 2017).

The rapidity with which visual responsiveness returned as a result of repeated exposure to cross-modal cues contrasts with the general intransigence of hemianopia under normal circumstances. Cross-modal events are a common feature of normal environments, and animals are likely to be exposed to thousands of such events in contralesional space every day. So, why is this “natural” multisensory exposure insufficient for rehabilitation while the laboratory exposure paradigm is so effective? One likely possibility is the difference in the density and regularity of the cross-modal events in these two circumstances. Cross-modal stimuli in the current rehabilitative paradigm were always congruent in space and time, and their individual physical features, spatiotemporal relationships, and iterative rates remained constant within and across exposure sessions. In a normal environment, a host of events gives rise to visual, auditory, and visual-auditory cues that can vary substantially in their physical features and in their cross-modal spatiotemporal relationships. Even repetition of the same event at different times in the non-laboratory environment is often accompanied by significant variation in the physical features and concordance of the cross-modal cues relative to the perceiver. Variation attributed to these and other sources can produce “contravening” experiences that may degrade the effectiveness of the stimuli in guiding underlying changes in the circuit.

The sensitivity of multisensory plasticity to regularity and congruency in cross-modal experience has been observed in other circumstances. Animals reared to adulthood in dark rooms, or with omnidirectional sound, have been deprived of the cross-modal experiences needed to develop the hallmark capability of normal SC neurons to integrate visual and auditory stimuli (Stein et al., 2014). Nevertheless, the later development of this capability in such animals can be rapidly initiated by repeatedly exposing them to these spatiotemporally congruent cross-modal stimuli (Yu et al., 2010; Xu et al., 2012). This is far less effectively initiated by “natural” sensory experience in normal environments (Rowland et al., 2014; Xu et al., 2017).

It is important to note that repeated exposure to such congruent cross-modal stimuli also amplifies the responses of SC neurons to their modality-specific component responses (Yu et al., 2009, 2013). In each of these cases, multisensory exposure is effective in this regard even when animals are anesthetized as they were here (Yu et al., 2009, 2010; Xu et al., 2012). The dependence of recovery on multisensory exposure, the required integrity of the AES-SC projection for this training to be effective (Jiang et al., 2015), the paucity of other visually-responsive structures in lesioned animals, and prior work showing that permanent hemianopia is induced when both cortex and SC are damaged (Sherman, 1977; Wallace et al., 1990), all point to the critical role of the SC in this recovery process. However, the specific neurological changes that enable the return of visual responsiveness in the SC remain to be determined.

Yet, particularly interesting is that overt visually-guided behavior returned in a central-to-peripheral progression despite the single 45° exposure site and that once recovery was initiated in central space, no additional training was required for it to extend throughout the entire contralesional visual field (see also Lomber et al., 2002). There are several factors that may have been involved. Many of the rehabilitated visually-responsive neurons were found to have receptive fields that extended into central visual space. Given that the SC has a very high density of neurons representing central visual space and a rapidly decreasing proportion representing more peripheral locations, the critical number of active neurons needed to support visual behavior may have been first achieved at more central locations and then successively at more peripheral locations. In addition, the neurons representing the most central region of visual space that were retained after the cortical lesion may have exaggerated this effect by exerting a bias on localization decisions that steadily weakened as visual responses returned in neurons representing more peripheral locations. These possibilities also require further exploration.

It should be noted that removing all contiguous areas of the visual cortex likely produces significant and permanent functional consequences that were not explored here. Physiological changes in SC neurons have been noted with visual cortex lesions of varying extent and these include limiting the capabilities of SC neurons to respond selectively to direction or velocity of movement, and lowering the incidence of binocularity (e.g., see McIlwain and Fields, 1971; Rosenquist and Palmer, 1971; Ogasawara et al., 1984; Hardy and Stein, 1988). Perceptually, it is likely that the lesion compromises higher-order visual functions such as those related to the identity or meaning of visual events.

Also important to note is that a region of ipsilateral association cortex (the AES) distant from the lesion site appears to play a crucial role in supporting the restored SC visual activity. Removing AES after training-induced recovery reinstates the hemianopia and eliminates SC visual responses (Jiang et al., 2015). This is the case despite the fact that the lesion of visual cortex also deprives AES of major sources of visual input (Mucke et al., 1982; Norita et al., 1986; Olson and Graybiel, 1987; Scannell et al., 1996), which would have initially minimized its visual contribution to the SC. However, there is a likely active reconfiguration and functional alteration in the capabilities of the remaining visual circuits (Payne et al., 1996; Sorenson and Rodman, 1999; Bridge et al., 2008; Das et al., 2012), possibly enhancing their visual inputs to AES and, in turn, the effect of AES on the SC.

A prime candidate for supplying the critical visual inputs for this role is the superficial SC (see also, Casagrande et al., 1972). Its neurons can access AES *via* thalamocortical relays (Mucke et al., 1982; Olson and Graybiel, 1987; Abramson and Chalupa, 1988; Harting et al., 1991; Kelly et al., 2003), can provide it with a rich source of visual information, and, as noted above, these SC neurons retain their visual responsiveness after the hemianopia-inducing lesion. Indeed, they are often thought to play a role in the residual (albeit unconscious) visuomotor capabilities of human patients referred to as “blindsight” (Leh et al., 2006, 2010; Cowey, 2010; Tamietto et al., 2010; but see Schmid et al., 2010). They are also believed to be involved in one of many functional loops in the nervous system which, in this context, could allow one part of the SC (the purely visual superficial layers) to provide functionally relevant input to another part (its multisensory layers) *via* AES (McHaffie et al., 2005). But, to use that visual input, or any other input that survives the lesion, such as the sparse projections from retina (Wässle and Illing, 1980), pretectum (Edwards et al., 1979; Huerta and Harting, 1982), or directly from the overlying superficial SC (Casagrande et al., 1972; Behan and Appell, 1992; Schnupp et al., 1995; King et al., 1998; May, 2006), the circuit must be sensitive to the multisensory exposure paradigm. Once again this points to the multisensory SC neuron itself and/or its local circuit as a primary locus of the rehabilitative effect. This is consistent with the observation that the characteristic capability of ME was possible in the few neurons examined. This capability may have already been present before the hemianopia was resolved *via* the combination of subthreshold visual and suprathreshold auditory inputs. Whether this is actually the case, and whether the process could impact perception and overt behavior at this time is currently unknown (but see Ten Brink et al., 2015).

Whatever combination of circuit changes was induced by the current exposure paradigm to restore visual responsiveness in the previously blind hemifield, the organismic variables generally thought to be important in learning and in functional recovery from brain damage were not essential in this context. It is also an open question about whether or not they could facilitate this process. Using alert, interactive, and rewarded animals in a previous study, Jiang et al. (2015) found that a similar cross-modal rehabilitative paradigm was effective after 11–12 days. Although this is half or less the exposure duration required here with the anesthetized animal, suggesting a facilitation effect, that exposure paradigm involved sessions 5 days/week, whereas exposure sessions (albeit, with a higher density of trials) were provided to the anesthetized animal only once/week. When measured in terms of the number of hours of “training” that led to rehabilitation, there was no obvious benefit of an alert behaving preparation. Although more controlled comparisons are clearly necessary before accepting what seems like a counterintuitive conclusion, the present findings do emphasize the sensitivity of the visual component of the multisensory circuit to the simple covariance of cross-modal cues. Repeated presentation of this stimulus complex led the circuit to regain many of its functional capabilities, thereby reversing hemianopia, and did so even when the host may have been unaware of the training experience.

## Data Availability Statement

The datasets generated for this study are available on request to the corresponding author.

## Ethics Statement

The methods were in compliance with the National Institutes of Health “Guide for the Care and Use of Laboratory Animals” (8th edition, NRC 2011) and approved by the Institutional Animal Care and Use Committee at Wake Forest School of Medicine.

## Author Contributions

HJ participated in the research design, data collection, analysis, and manuscript writing. BR and BS participated in the research design, analysis, and manuscript writing.

## Conflict of Interest

The authors declare that the research was conducted in the absence of any commercial or financial relationships that could be construed as a potential conflict of interest.
